# MicroRNA Signatures in Malignant Pleural Mesothelioma Effusions

**DOI:** 10.1155/2019/8628612

**Published:** 2019-07-31

**Authors:** Kimberly A. Birnie, Cecilia M. Prêle, Arthur W. (Bill) Musk, Nicholas de Klerk, Y. C. Gary Lee, Deirdre Fitzgerald, Richard J. N. Allcock, Philip J. Thompson, Jenette Creaney, Bahareh Badrian, Steven E. Mutsaers

**Affiliations:** ^1^Institute for Respiratory Health and Centre for Respiratory Health, School of Biomedical Sciences, University of Western Australia, Nedlands, WA 6009, Australia; ^2^Centre for Cell Therapy and Regenerative Medicine, School of Biomedical Sciences, University of Western Australia and Harry Perkins Institute of Medical Research, Nedlands, WA 6009, Australia; ^3^Occupational Respiratory Epidemiology, School of Population and Global Health, University of Western Australia, Crawley, WA 6009, Australia; ^4^Department of Respiratory Medicine, Sir Charles Gairdner Hospital, Nedlands, WA 6009, Australia; ^5^Telethon Kids Institute and Centre for Child Health Research, University of Western Australia, Nedlands, WA 6009, Australia; ^6^School of Biomedical Sciences, University of Western Australia, and Pathwest Laboratory Medicine, QEII Medical Centre, Nedlands, WA 6009, Australia; ^7^Pathwest Laboratory Medicine WA, QEII Medical Centre, Monash Avenue, Nedlands, WA 6009, Australia; ^8^Medical School, QEII Medical Centre, University of Western Australia, Nedlands, WA 6009, Australia

## Abstract

Malignant pleural mesothelioma (MPM) is an incurable cancer of the pleura that can be difficult to diagnose. Biomarkers for an easier and/or earlier diagnosis are needed. Approximately 90% of MPM patients develop a pleural effusion (PE). PEs are ideal sources of biomarkers as the fluid would almost always require drainage for diagnostic and/or therapeutic reasons. However, differentiating MPM PE from PE caused by other diseases can be challenging. MicroRNAs are popular biomarkers given their stable expression in tissue and fluid. MicroRNAs have been analysed in PE cytology samples for the diagnosis of MPM but have not been measured in frozen/fresh PE. We hypothesise that microRNAs expressed in PE are biomarkers for MPM. TaqMan OpenArray was used to analyse over 700 microRNAs in PE cells and supernatants from 26 MPMs and 21 other PE-causing diseases. In PE cells, combining miR-143, miR-210, and miR-200c could differentiate MPM with an area under the curve (AUC) of 0.92. The three-microRNA signature could also discriminate MPM from a further 40 adenocarcinomas with an AUC of 0.9887. These results suggest that the expression of miR-143, miR-210, and miR-200c in PE cells might provide a signature for diagnosing MPM.

## 1. Introduction

Malignant pleural mesothelioma (MPM) is an aggressive tumour with an incidence expected to increase over the next 10-20 years [1]. MPM is largely unresponsive to conventional therapy, and patient prognosis is often <12 months. A long latency period and the advanced age of most patients make MPM even more difficult to treat. A major limitation to the management of MPM is the difficulty in obtaining a definitive and early diagnosis [2].

Patients often present with breathing difficulties caused by accumulation of fluid in the pleural space. Pleural effusions (PE) can be cytologically examined to diagnose MPM. However, the sensitivity of a cytological diagnosis is somewhat lower than that of a histological diagnosis [3]. Hence, most clinics rely on tissue biopsy to diagnose MPM. Given the vast surface area of the parietal pleura, a sampling error from percutaneous biopsy can occur. Therefore, patients are often subjected to invasive thoracoscopy or thoracotomy to obtain tissue from multiple sites. The costs, delay, and adverse events associated with this approach are well recognised [4]. In addition, MPM can mimic adenocarcinomas originating from, or that metastasise to, the lung [5]. Staining tissue with antibodies such as calretinin, podoplanin WT1, CEA, Ber-EP4, and MOC31 may indicate if the malignancy is of mesothelial or epithelial origin; however, no antibody alone is specific for either type and false positives can occur [6]. Therefore, clinical and imaging data are often also required to confirm a diagnosis of MPM [3].

The discovery of a relatively noninvasive diagnostic marker with high positive/negative predictive values would represent a breakthrough in MPM care. Previous studies have screened patient serum and PE for novel biomarkers [7] with soluble mesothelin and fibulin-3 the most promising [8–10]. However, limitations exist [2].

MicroRNAs are powerful regulators of gene expression and potential therapeutic and diagnostic targets for cancer. The short, endogenous, noncoding RNAs regulate genes by repressing translation or promoting mRNA degradation [11]. MicroRNAs are attractive targets for biomarker analysis due to their stability within the body and stored samples [12]. MicroRNAs have been evaluated for this purpose in MPM tissue, serum/plasma, the cellular fraction of peripheral blood, cell lines, and archived cytology samples [13, 14]. However, results often vary and large patient cohorts to assess clinical relevance are needed. Furthermore, the diagnostic potential of microRNA within fresh/frozen PEs has not been analysed. We hypothesise that microRNAs expressed in PEs are biomarkers for MPM. To address this hypothesis, we analysed microRNA by TaqMan OpenArray and quantitative real-time PCR (qRT-PCR) in PE cells and supernatants from patients with MPM, various adenocarcinomas, and benign diseases. A three-microRNA signature was identified in PE cells for differentiating MPM from adenocarcinoma and benign patients.

## 2. Materials and Methods

### 2.1. Patient Samples

The collection and use of PEs were approved by the Sir Charles Gairdner Hospital Human Research Ethics Committee, Perth, Western Australia. MPM was diagnosed by a pathologist based on effusion cytology with ancillary immunohistochemical stains. In a third of cases, histological samples confirm diagnosis. A clinical review was also performed to supplement the pathological review (YCGL or DF) and classified as being caused by MPM, an adenocarcinoma, or a benign disease.

Clinicopathological features of patients are shown in Table 1. PE samples analysed were the earliest available from each patient. TaqMan OpenArray profiling was performed on 26 MPMs, 11 adenocarcinomas, and 10 benign samples randomly selected from a biobank. These PEs were processed at room temperature within 12 hours of collection. Cells were harvested by centrifuging the PEs (up to 300 mL) at 1020 x g for 15 minutes (min). Red blood cells were lysed by incubating pellets with 1x red blood cell lysis buffer (BioLegend, San Diego, California) for 5 min. The supernatants (3 mL) and cells resuspended in QIAzol (Qiagen, Germantown, Philadelphia) were stored at -80°C. PE cell samples from another 40 adenocarcinomas were randomly selected from the Australian Mesothelioma Tissue Bank, Perth, Western Australia. The cells were collected by centrifuging the PEs at 1020 x g for 10 min, removing the supernatant, and resuspending the cells in 1 mL of Ultraspec (amsbio, Abingdon, United Kingdom). Samples were rested on ice for 30 min and stored at -80°C.

### 2.2. RNA Isolation

Total RNA was extracted from cells using the miRNeasy Kit (Qiagen) according to the manufacturer's protocol. An additional step of DNase treatment with 80 *μ*L of Rnase-Free DNAse solution (Qiagen) was added prior to column elution. Total RNA was extracted from supernatants using a miRVana PARIS Kit (Life Technologies, Mulgrave, Australia) according to the manufacturer's protocol following a double phenol-chloroform (Life Technologies) extraction. Eluted RNA was quantitated using a Nanodrop ND1000 spectrophotometer (Thermo Fisher Scientific, Waltham, Massachusetts).

### 2.3. TaqMan OpenArray

Reverse transcription, preamplification, and TaqMan OpenArray (Life Technologies) were carried out according to the manufacturer's protocol using either pool A or pool B Megaplex primers.

### 2.4. Quantitative Real-Time PCR (qRT-PCR)

Total RNA (10 ng) was reverse transcribed using the microRNA TaqMan Reverse Transcription Kit (Life Technologies) and analysed by qRT-PCR with TaqMan primer probes (Life Technologies) as previously described [15]. Mesothelin and fibulin-3 mRNA were measured using TaqMan primer probes with PGK-1 as a housekeeping control (Life Technologies).

### 2.5. Statistical Analysis

OpenArray data was analysed using DataAssist 3.0™ (Life Technologies). MicroRNAs with a cycle threshold (CT) of 30 or more were considered unamplified. Expression relative to the average expressions of the endogenous controls RNU44 and RNU48 was determined using the 2^–*Δ*CT^ method. RNU44 and RNU48 were chosen for normalisation due to low standard deviations in expression. MicroRNAs were determined to be significantly up- or downregulated in MPM with a twofold change and a *p* value ≥ 0.05 after adjusting for false discovery using the Benjamin Hochberg method. Results were also analysed using GraphPad Prism 4 (GraphPad Software Inc., La Jolla, California). The statistical significance was determined using Student's *t*-test or a Mann-Whitney test for data that was not normally distributed. Logistic regression was used to analyse combinations of microRNA with the microRNA values (logged) as predictors using Stata version 14.2 (Stata Corporation, College Station, Texas). Receiver operating characteristic (ROC) curves and the area under the curves (AUC) were calculated to assess diagnostic ability. We also report sensitivity, specificity, positive, and negative predictive values.

## 3. Results

### 3.1. MicroRNAs Are Differentially Expressed in MPM PE Cells

To identify novel diagnostic targets for MPM, 758 microRNAs and controls were analysed by TaqMan OpenArray in 47 PE cells and supernatants (Table 1). Among the MPM patients (*n* = 26), 88% were male and 65% were diagnosed with the epithelioid subtype. Patients with a benign disease (*n* = 10) were mostly males (70%) and spread across six classifications. The adenocarcinoma patients (*n* = 11) included five lung, five breast, and one ovarian cancers. Of these, 73% were female. Patient ages in the three disease cohorts were statistically similar.

**Figure 1:**
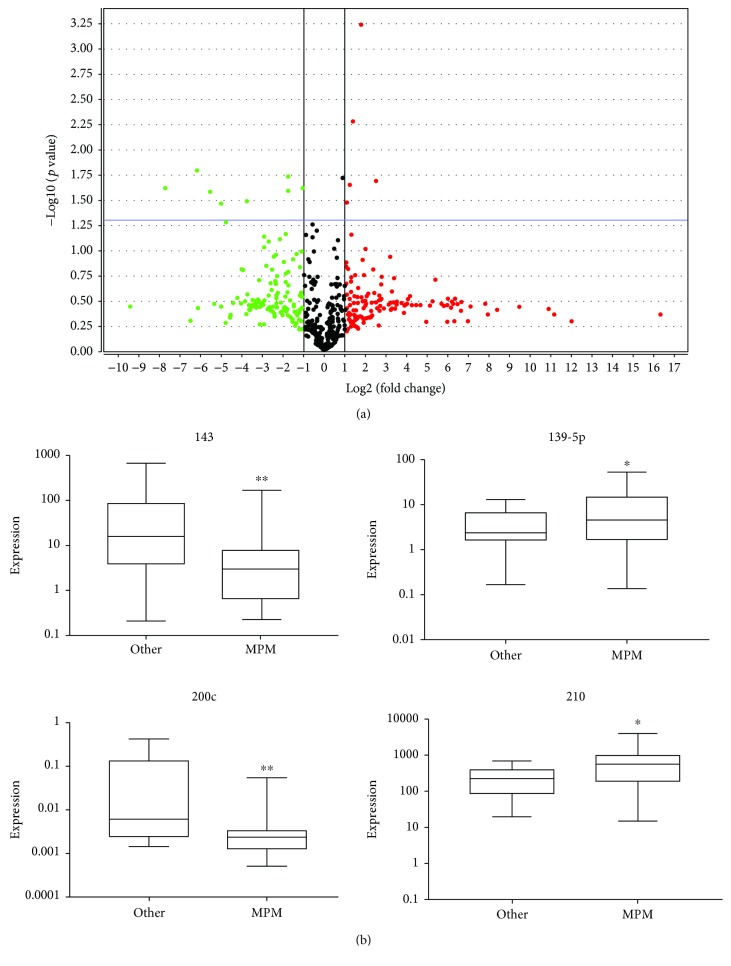
MicroRNAs are differentially expressed in MPM PE cells. (a) Volcano plot of significantly up- (red dots) and downregulated (green dots) microRNA in MPM vs. other diseases (adenocarcinoma and benign diseases combined) as determined by TaqMan OpenArray profiling. (b) Expression of the top differentially expressed microRNA between MPM and other PE-causing diseases as validated by RT-qPCR. MicroRNA expression was normalised to RNU44 and 48, expressed as 2^–*Δ*CT^, and plotted on a logarithmic scale. The line within the boxes represents the median values, and the top and bottom of the boxes indicate the interquartile ranges. The whiskers demonstrate the upper and lower adjacent values for each disease group (^∗∗^ *p* < 0.01, ^∗^ *p* < 0.05).

A volcano plot of the OpenArray data shows microRNAs that were significantly up- or downregulated in MPM compared to other diseases (adenocarcinoma and benign pleural disease combined) (Figure 1(a)). In MPM PE cells, seven microRNAs were significantly downregulated (miR-874, miR-31, miR-203, miR-200a, miR-143, miR-200c, and miR-200b) and four significantly upregulated (miR-139-5p, miR-210, miR-944, and miR-320) (Table 2). Two of the downregulated microRNAs (mir-143 and mir-200c) and two of the upregulated microRNAs (miR-139-5p and miR-210) were validated by qRT-PCR in the same sample set (*p* < 0.05, Figure 1(b)). ROC curves were generated to compare the ability of each microRNA to individually differentiate MPM from the other diseases (Table 3). MiR-200c was the best discriminator with an AUC of 0.79 (95% CI: 0.66, 0.92) and an odds ratio of -0.87 (95% CI: -1.49, -0.24).

OpenArray analysis of PE supernatants revealed a small number of microRNAs expressed significantly different between MPM, adenocarcinoma, and benign diseases. These microRNAs (miR-186, miR-29a, miR-31, and miR-342-3p) were also measured by qRT-PCR, and the results did not confirm the OpenArray expression profiles (Supplementary Figure 1). Therefore, the microRNAs identified in the PE supernatants are unlikely to be useful biomarkers for diagnosing MPM.

### 3.2. Combining miR-143, miR-210, and miR-200c Provided a Signature for Differentiating MPM from Adenocarcinoma and Patients with a Benign Disease

**Figure 2:**
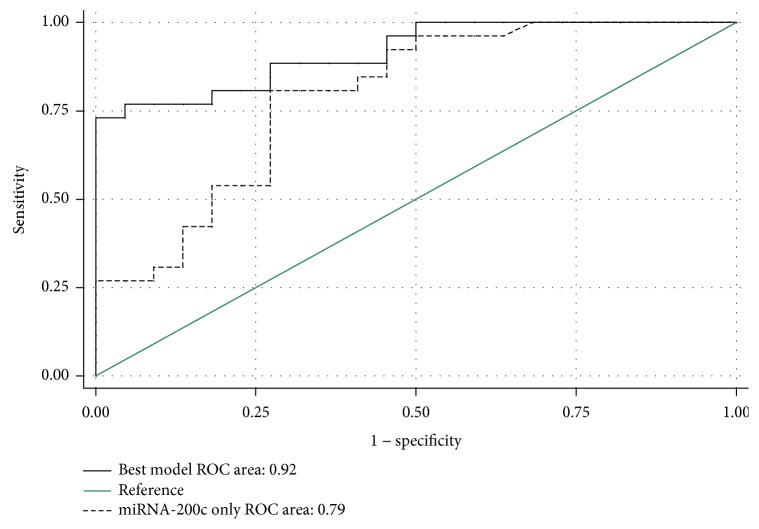
The combination of miR-200c, miR-210, and miR-143 was best for discriminating MPM from other PE-causing diseases. Using the three-microRNA signature to differentiate MPM from other PE-causing diseases was assessed by generating a ROC curve. The ROC curve for miR-200c is included for comparison.

The microRNAs identified as differentially expressed in MPM PE cells were moderate discriminators between MPM and the other PE-causing diseases. To improve diagnostic efficiency, logistic regression was used to assess combinations of miR-143, miR-210, miR-200c, and miR-139-5p. Following ROC curve analysis, the combination of miR-200c, miR-210, and miR-143 provided an AUC of 0.92 (95% CI: 0.84, 0.99) (Table 4 and Figure 2). The three-microRNA signature was significantly better at differentiating MPM compared to any of the microRNAs alone (Table 3).

### 3.3. Combining Fibulin-3 with miR-143, miR-210, and miR-200c Did Not Increase Diagnostic Efficiency

**Figure 3:**
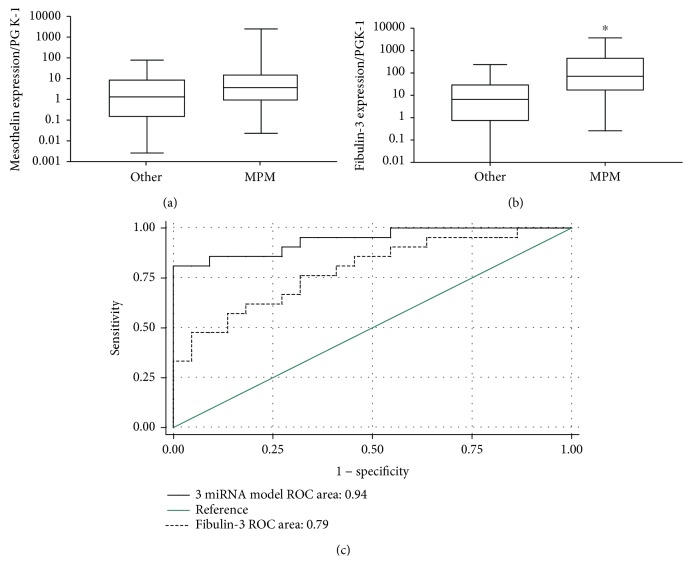
Fibulin-3 mRNA is expressed significantly higher in MPM PE cells. (a) Mesothelin and (b) fibulin-3 mRNA were measured in cells isolated from PE using qRT-PCR. Expression was plotted on a logarithmic scale, and PGK-1 was used as an endogenous control. The line within the boxes represents the median values, and the top and bottom of the boxes indicate the interquartile ranges. The whiskers demonstrate the upper and lower adjacent values for each disease group (^∗^ *p* < 0.05). (c) The efficiency of using fibulin-3 to differentiate MPM from other PE-causing diseases was assessed using ROC curve analysis and expressed as the AUC. The ROC curve for miR-200c, miR-210, and miR-143 in the same samples is included for comparison.

Soluble mesothelin and fibulin-3 are potential biomarkers for MPM that were previously reported as highly expressed in the MPM PE supernatant [10, 16]. In the current study, the three-microRNA signature was compared to levels of mesothelin and fibulin-3 mRNA in PE cells using qRT-PCR. Five MPM samples were excluded from the analysis due to a lack of remaining RNA. Mesothelin mRNA levels were not significantly different between the patient groups whereas fibulin-3 was expressed significantly higher in MPM (Figures 3(a) and 3(b)). Fibulin-3 was found to be a strong predictor for a diagnosis of MPM with an AUC of 0.79 (95% CI: 0.65, 0.93) (Figure 3(c)). The addition of fibulin-3 to miR-143, miR-210, and miR-200c did not improve the diagnostic efficiency of the signature. In the reduced sample set, the microRNA model could differentiate MPM from the other diseases with an AUC of 0.94 (95% CI: 0.87, 1.00) (Figure 3(c)).

### 3.4. MiR-143, miR-210 and miR-200c, Provided a Signature for Differentiating MPM from a Range of PE Causing Adenocarcinomas

**Figure 4:**
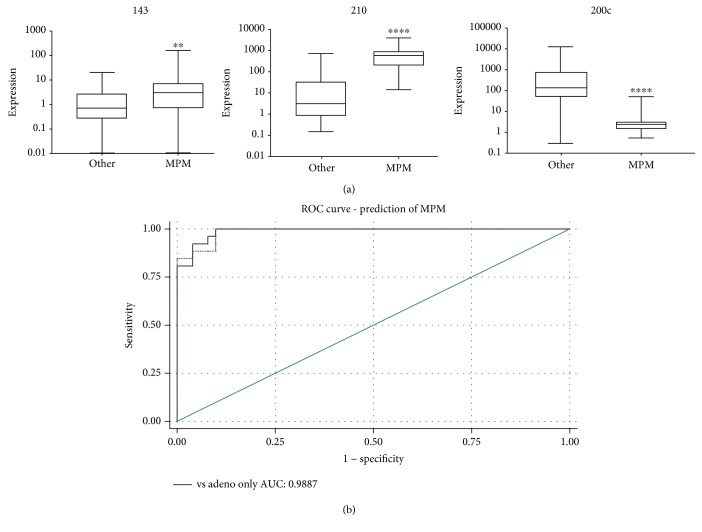
The combination of miR-200c, miR-210, and miR-143 could discriminate MPM from a range of adenocarcinomas. (a) The expression of miR-143, miR-200c, and miR-210 in MPM and a range of adenocarcinomas were measured by qRT-qPCR. MicroRNA expression was normalised to RNU44 and 48, expressed as 2^–*Δ*CT^, and plotted on a logarithmic scale. The line within the boxes represents the median values, and the top and bottom of the boxes indicate the interquartile ranges. The whiskers demonstrate the upper and lower adjacent values for each disease group (^∗∗^ *p* < 0.01, ^∗∗∗^ *p* < 0.001, and ^∗∗∗∗^ *p* < 0.0001). (b) Using the three-microRNA signature to differentiate MPM from adenocarcinomas was assessed by generating a ROC curve.

To determine if the three-microRNA signature can distinguish MPM from a range of PE-causing adenocarcinomas, we measured miR-143, miR-200c, and miR-210 by qRT-PCR in an additional 40 PE cell adenocarcinoma samples and compared the microRNA expression levels to those detected in MPM PE cells. The adenocarcinoma samples were derived from patients with breast, ovarian, pancreatic, colon, and unknown malignancies (Table 1). All three microRNAs were expressed significantly different in MPM compared to the adenocarcinomas (Figure 4(a)). Interestingly, the expression of miR-143 in Figure 4(a) is higher in MPM than in control whereas in Figure 1(b) the expression is lower in MPM compared with control. The discrepancy is likely due to the makeup of the controls. In Figure 1(b), the controls consisted predominately of benign pleural diseases with some lung and breast adenocarcinomas whereas in Figure 4 the controls were all adenocarcinomas. Future validation studies need to address this discrepancy.

The combination of miR-200c, miR-210, and miR-143 could differentiate MPM from adenocarcinoma patients with an AUC of 0.9887 (95% CI: 0.97377, 1.00000) (Figure 4(b)), a sensitivity of 92.31%, a specificity of 96.08%, a positive predictive value of 92.31%, and a negative predictive value of 96.08%. Overall, 94.81% of the samples were correctly classified using the microRNA model.

## 4. Discussion

MPM can be difficult to differentially diagnose, particularly from diseases that also cause PE [17]. PEs are attractive sources of biomarkers for MPM, and staining PE cells with a panel of antibodies can assist a diagnosis [3]. However, this can be expensive, sample consuming, and affected by subjective interpretation. This is further complicated by factors such as establishing whether asbestos exposure had occurred and limitations associated with imaging analysis. To overcome these issues, patient PE and serum have been analysed to identify noninvasive biomarkers for MPM. Some of the most extensively evaluated markers such as osteopontin [18, 19], fibulin-3 [10], and soluble mesothelin [16, 20] show promise, but limitations remain [2].

MicroRNAs have become attractive targets as novel biomarkers for MPM [13, 14, 21–23]. MicroRNAs can be easily quantitated in a range of tissue types in an unbiased manner. However, MPM studies report variable results from analyses largely focused on tissue collected from small patient cohorts. More recently, the diagnostic potential of microRNAs in PE cytology specimens was investigated. Complementary to a cytological analysis, miR-21 and miR-126 were identified for differentiating MPM from reactive mesothelial cells [14] and miR-130a for differentiating MPM from lung adenocarcinoma [24]. However, the performance of miR-130a was found to be no better than an immunohistochemical diagnosis, [24] and because only a select number of microRNAs were analysed, more efficient biomarkers could have been overlooked [14]. Whether frozen/fresh PE microRNAs can be used to diagnose MPM is yet to be determined. Therefore, we aimed to analyse the microRNAome in PE cells and supernatants to identify targets for differentiating MPM from other PE-causing diseases.

Using TaqMan OpenArray, we identified microRNAs expressed significantly different in PE cells from MPM, benign, and adenocarcinoma patients. The combination of miR-143, miR-210, and miR-200c provided a signature that could differentiate MPM from other PE-causing diseases with an AUC of 0.92–0.98. The International Mesothelioma Interest Group recommends good biomarkers have sensitivity and specificities above 80% [25]. The microRNA signature we identified had characteristics much higher than this threshold and when compared to previously reported PE biomarkers fibulin-3 [9] and soluble mesothelin [8].

We also measured mesothelin and fibulin-3 mRNA in PE cells, and whilst mesothelin levels were the same in all disease cohorts, fibulin-3 was expressed significantly higher in MPM. Fibulin-3 could differentiate MPM from the other PE-causing diseases with an AUC similar to what was previously reported (0.83) [9]. Combining fibulin-3 with miR-143, miR-210, and miR-200c did not improve the diagnostic efficiency of the signature. It is important to note that soluble mesothelin and fibulin-3 protein, not mRNA, have been demonstrated as PE biomarkers for MPM [8–10]. Therefore, measuring soluble mesothelin and fibulin-3 in PE supernatants may provide a biomarker that can be combined with the microRNA to improve the diagnostic efficiency of the signature.

Analysing PE cells for microRNA biomarkers is advantageous because high concentrations of quality RNA can be isolated, and a robust analysis performed. However, various cell populations exist within PE; thus, the microRNAs we identified may not be specific for MPM cells. However, diagnostic tests need to be simple and easy to perform and separating cell populations to obtain purely malignant cells does not fit this description. The signature we identified may also not be suitable for diagnosing all MPM subtypes as usually only epithelioid MPM cells are shed into the pleural space.

In agreement with the results reported in this study, miR-143, miR-210, and miR-200c were previously shown to be aberrantly expressed in MPM tissue [26–28]. These microRNAs may be biologically important in MPM as they are predicted to regulate signalling pathways (ERK5 [29], EGFR [30], Bcl-2 [31, 32], and Wnt [33]), known to play a role in this disease. Therefore, miR-143, miR-210, and miR-200c may also be novel therapeutic targets for MPM.

PE fluid is in direct contact with MPM cells and is a source of microRNA released from these cells. Therefore, we also analysed the PE supernatant microRNA profiles. These profiles appeared to be significantly different between the disease cohorts following OpenArray analysis. However, the results did not validate when the microRNAs were measured by qRT-PCR. The samples had a variety of physical characteristics including a range of viscosities that likely contributed to a reduced RNA extraction efficiency and impacted on downstream analysis. MicroRNAs have been isolated and measured in PE fluid [34, 35]; however, results between studies are highly variable, with no standardised protocol available. For better consistency within and between studies, a robust procedure needs to be developed.

## 5. Conclusion

Despite not being able to identify PE supernatant biomarkers for MPM, we have shown that miR-200c, miR-210, and miR-143 are potential PE cell biomarkers for differentiating MPM from benign and malignant PE-causing diseases. The microRNA signature requires testing in a larger patient cohort, and given that MPM is a relatively rare tumour, this likely needs to be a multicentre collaboration. Developing an easier and faster diagnostic test for MPM may facilitate earlier treatment and improve patient outcomes.

## Figures and Tables

**Table 1:** Patient characteristics.

Diagnosis	*n*	Age ± SD	Gender	
			Male	Female
*MPM*				
Epithelioid	17	70.8 (±8.9)	14	3
Sarcomatoid	2	69.5	2	—
Biphasic	1	76	1	—
Desmoplastic	1	82	1	—
Unspecified	5	67 (±8.7)	5	—
*Benign*				
Parapneumonic effusion	2	52.6	2	—
Hepatic hydrothorax	1	68.0	—	1
Pleuritis	1	84.0	1	—
Trapped lung	1	74.0	1	—
Renal transudate	1	64.0	1	—
Unspecified	4	60.0 (±25.5)	2	2
*Metastatic adenocarcinoma*				
Cohort 1				
Lung	5	65.2 (±30.8)	3	2
Breast	5	66.2 (±12.8)	—	5
Ovarian	1	90	—	1
Cohort 2				
Colon	3	63.6 (±11.6)	3	—
Breast	13	62.5 (±12.6)	—	13
Ovarian	11	60.2 (±10.3)	—	11
Pancreatic	4	70.2 (±4.99)	3	1
Other	7	65.6 (±9.24)	5	2
Unknown primary	2	80	1	1

**Table 2:** MicroRNA up- and downregulated in MPM PE cells compared to controls (adenocarcinoma and benign pleural diseases combined) as determined by OpenArray.

Down			Up		
miRNA	Fold change	*p* value	miRNA	Fold change	*p* value
miR-200b	0.004	0.023	miR-944	5.700	0.2020
miR-200c	0.013	0.010	miR-139-5p	3.418	0.0057
miR-143	0.020	0.026	miR-210	2.590	0.0052
miR-200a	0.030	0.034	miR-320	2.380	0.0220
miR-203	0.074	0.032			
miR-31	0.298	0.012			
miR-874	0.4818	0.023			

**Table 3:** Log odds ratios (OR) for each microRNA used to differentiate MPM from controls (adenocarcinoma and benign combined).

miRNA	Log (OR)	95% CI	AUC	95% CI	*p* value
miR-210	0.59	0.07, 1.11	0.72	0.58, 0.87	0.03
miR-143	-0.30	-0.62, 0.01	0.66	0.50, 0.82	0.06
miR-200c	-0.87	-1.49, -0.24	0.79	0.66, 0.92	0.006
miR-139-5p	0.42	-0.01, 0.85	0.65	0.50, 0.81	0.06

**Table 4:** Log OR for the combined microRNA model used to differentiate MPM from controls (adenocarcinoma and combined).

miRNA	Log (OR)	95% CI	*p* value	AUC	95% CI
miR-210	0.99	0.18, 1.79	0.017	0.92	0.84, 0.99
miR-143	-0.66	-1.16, -0.17	0.008		
miR-200c	-1.40	-2.32, -0.48	0.003		
Constant	-1.41	-3.81, 0.98			

## Data Availability

Data enquiries can be addressed to the corresponding author Associate Professor Steven Mutsaers, Institute for Respiratory Health, 5th Floor Harry Perkins Institute of Medical Research, QEII Medical Centre, 6 Verdun Street, Nedlands, WA, 6009, Australia (email: steven.mutsaers@uwa.edu.au).

## Supplementary Materials

Supplementary Materials Figure S1: potential microRNA biomarkers used for differentiating MPM from other PE 469-causing diseases were identified in PE supernatants by OpenArray profiling. The expression 470 profiles for (a) miR-186, (b) miR-29a, (c) miR-31, and (d) miR-342-3p did not validate when 471 microRNAs were measured by qRT-PCR. MicroRNA expression was plotted on a 472 logarithmic scale, and U6snRNA was used as an endogenous control. The line within the 473 boxes represents the median values, and the top and bottom of the boxes indicate the 474 interquartile ranges. The whiskers demonstrate the upper and lower adjacent values for each 475-sample group (^∗∗^ *p* < 0.01, ^∗^ *p* < 0.05).
