# Supplementary material for: MicroRNA Signatures in Malignant Pleural Mesothelioma Effusions
Source: Dis Markers. 2019 Jul 31;2019:8628612. doi: 10.1155/2019/8628612 (PMC6701424; doi:10.1155/2019/8628612)
Supplement: Supplementary Materials — Figure S1: potential microRNA biomarkers used for differentiating MPM from other PE 469-causing diseases were identified in PE supernatants by OpenArray profiling. The expression 470 profiles for (a) miR-186, (b) miR-29a, (c) miR-31, and (d) miR-342-3p did not validate when 471 microRNAs were measured by qRT-PCR. MicroRNA expression was plotted on a 472 logarithmic scale, and U6snRNA was used as an endogenous control. The line within the 473 boxes represents the median values, and the top and bottom of the boxes indicate the 474 interquartile ranges. The whiskers demonstrate the upper and lower adjacent values for each 475-sample group (∗∗ p < 0.01, ∗ p < 0.05). [file 8628612.f1.pdf]

Supplementary Files:

Figure S1. Potential microRNA biomarkers used for differentiating MPM from other PE causing diseases were identified in PE supernatants by OpenArray profiling. The expression profiles for (a) miR-186, (b) miR-29a, (c) miR-31 and (d) miR-342-3p did not validate when the microRNAs were measured by qRT-PCR. MicroRNA expression was plotted on a logarythmic scale and U6snRNA was used as an endogenous control. The line within the boxes represents the median values and the top and bottom of the boxes indicates the interquartile ranges. The whiskers demonstrate the upper and lower adjacent values for each sample group. ** $p< 0.01$. * $p< 0.05$.

Figure s1

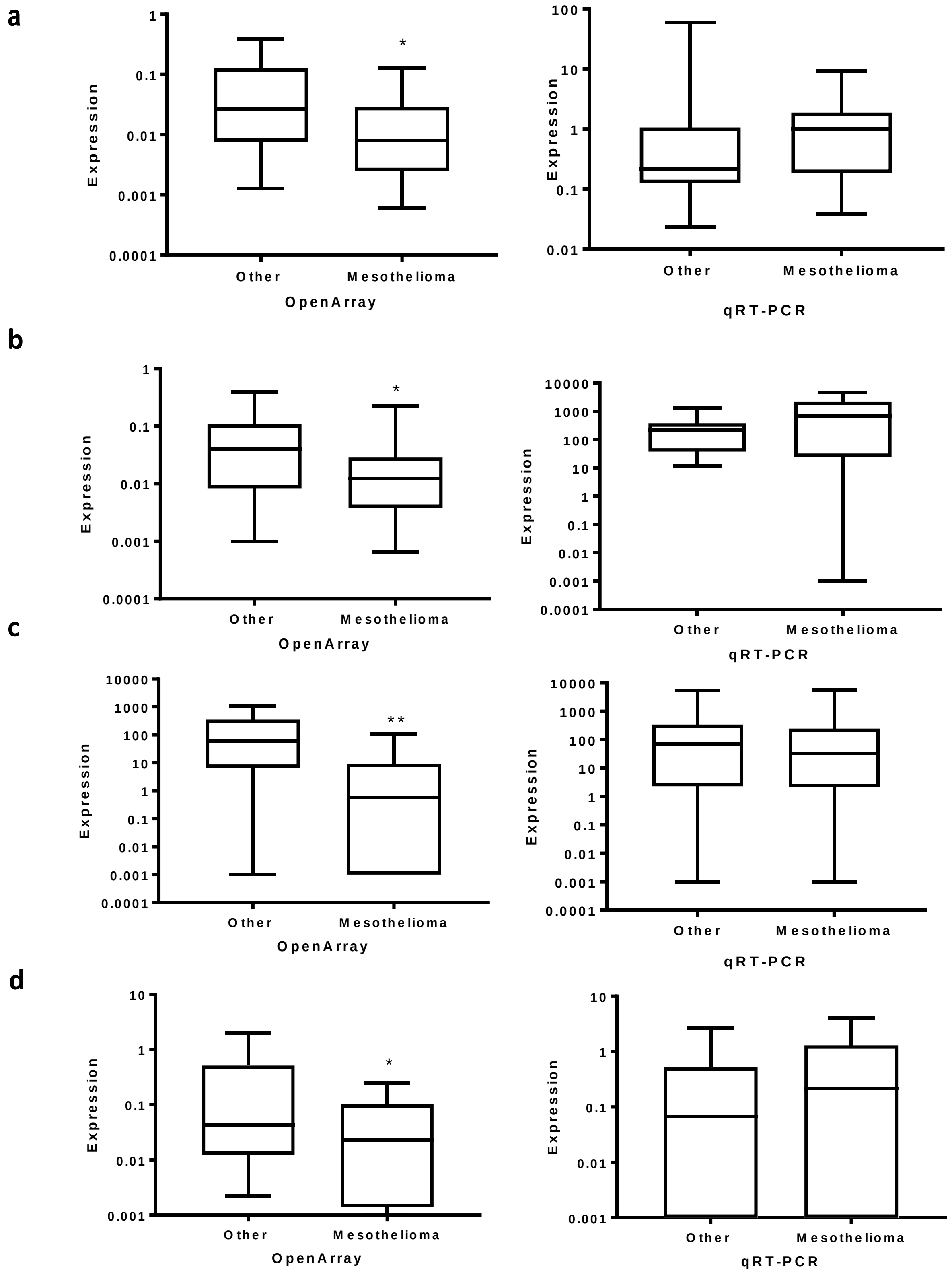
a
b
c
d
Expression
Other
Mesothelioma
OpenArray
qRT-PCR
*
**
1
0.1
0.01
0.001
0.0001
100
10
10000
1000
